# Cervical spine reposition errors after cervical flexion and extension

**DOI:** 10.1186/s12891-017-1454-z

**Published:** 2017-03-13

**Authors:** Xu Wang, René Lindstroem, Niels Peter Bak Carstens, Thomas Graven-Nielsen

**Affiliations:** 1 SMI, Department of Health and Science Technology, Faculty of Medicine, Aalborg University, Fredrik Bajers Vej 7D-3, 9220 Aalborg E, Denmark; 2 Vejgaard Kiropraktisk Klinik, Aalborg, Denmark; 3 Department of Orthopedics, The Second Hospital of Jilin University, Ziqiang 218, Changchun, 130041 Jilin People’s Republic of China; 4 Center for Neuroplasticity and Pain (CNAP), SMI, Department of Health and Science Technology, Faculty of Medicine, Aalborg University, Fredrik Bajers Vej, 7D-39220 Aalborg, Denmark

**Keywords:** Spine, Neck, Fluoroscopy, Cervical vertebrae, Position sense, Motion, Flexion and extension, Repositioning

## Abstract

**Background:**

Upright head and neck position has been frequently applied as baseline for diagnosis of neck problems. However, the variance of the position after cervical motions has never been demonstrated. Thus, it is unclear if the baseline position varies evenly across the cervical joints. The purpose was to assess reposition errors of upright cervical spine.

**Methods:**

Cervical reposition errors were measured in twenty healthy subjects (6 females) using video-fluoroscopy. Two flexion movements were performed with a 20 s interval, the same was repeated for extension, with an interval of 5 min between flexion and extension movements. Cervical joint positions were assessed with anatomical landmarks and external markers in a Matlab program. Reposition errors were extracted in degrees (initial position minus reposition) as constant errors (CEs) and absolute errors (AEs).

**Results:**

Twelve of twenty-eight CEs (7 joints times 4 repositions) exceeded the minimal detectable change (MDC), while all AEs exceeded the MDC. Averaged AEs across the cervical joints were larger after 5 min’ intervals compared to 20 s intervals (*p* < 0.05).

**Conclusions:**

This is the first study to demonstrate single joint reposition errors of the cervical spine. The cervical spine returns to the upright positions with a 2° average absolute difference after cervical flexion and extension movements in healthy adults.

**Electronic supplementary material:**

The online version of this article (doi:10.1186/s12891-017-1454-z) contains supplementary material, which is available to authorized users.

## Background

The upright head and neck position is the most frequent human posture of daily life. This position is baseline for scientific studies and diagnosis [1–3]. The natural head position can be compared within or between subjects [4], and the initial head position may influence movements of the cervical spine [5, 6]. The variation of the upright posture after neck movements between cervical joints or cervical regions are unknown, and it is unclear if cervical spine motion should be regarded in single joint units, as multi-joint units or as regional units with respect to joint reposition.

Cervical x-rays include the upright posture, and change in flexion and extension x-rays are assessed from the upright neck posture. Such as in pre- and post-surgical evaluation of cervical spine motions [1–3, 7–9]. Knowledge of the variance in cervical upright joint repositioning, is a prerequisite for assessment of dynamic cervical joint motion; however, the variance has never been investigated. Thus, it is unclear how much the upright neck varies in scientific investigation and diagnosis.

Impaired proprioception has been demonstrated in patients with cervical disorders and forward head postures [10–14]. Cervical radiculopathy patients showed impaired head reposition acuity compared with healthy controls [2]. Head or neck repositioning were also impaired in older adults [15], and patients with cervical spondylosis [16], cervicogenic dizziness [17], whiplash [18, 19], muscle fatigue [20], and non-traumatic neck pain [21–23]. In general, reposition acuity has been used to evaluate proprioception [24]. Subjects with pain in the upper cervical region demonstrated additional impairment in sensorimotor control compared to subjects with pain in the lower cervical region [21].

Head and neck positions can be resolved with respect to the horizontal plane or other anatomical structures [2, 8]. For both assessments it is unknown if head and neck reposition errors mainly occur in the suboccipital region or in the cervical regions below.

The sub-occipital anatomy, muscle density and function are different compared with that of the lower cervical spine [25]. The distinct osseous shape*s* of occiput, atlas, and axis underlie functional differences. The upper cervical spine contributes with almost 60% of free and unrestricted cervical spine axial rotation [26].

The acuity of cervical joints’ position sense in healthy subjects is important. This is because reposition errors of the upright position are reflected in dynamic motion of the cervical spine and in clinical studies, where the upright cervical spine serves as baseline [6].

Memory of position or time delay effects influence the cervical joint position sense [27, 28]. Time delay is hypothesized to affect the reposition of cervical joints. However, the effect of time delay on neck reposition is unknown.

The aim of this study was to assess healthy cervical spine reposition errors in the upright position. It was hypothesized that 1) all single cervical joints demonstrate reposition errors between end-range flexion and extension movements and 2) the reposition error is increased with longer time delays.

## Methods

### Participants

Six healthy females (age: 24.3 ± 3.8 years; height: 163.5 ± 6.0 cm; weight: 56.8 ± 7.5 kg; body mass index: 21.2 ± 2.4 kg/m^2^; mean ± standard deviation) and 14 healthy males (27.6 ± 5.4 years; 179.1 ± 6.6 cm; 74.1 ± 6.6 kg; 23.0 ± 1.5 kg/m^2^) without neck symptoms within the last 3 months were included. Exclusion criteria were possible pregnancy and any neck disorder. All participants were recruited from university students and staff through bulletins and a website.

### Ethics, consent and permissions

The study was conducted in accordance with the Declaration of Helsinki and approved by the North Denmark Region ethics committee (N20140004). Participants signed a written informed consent form.

### Experimental procedure

**Fig. 1:**
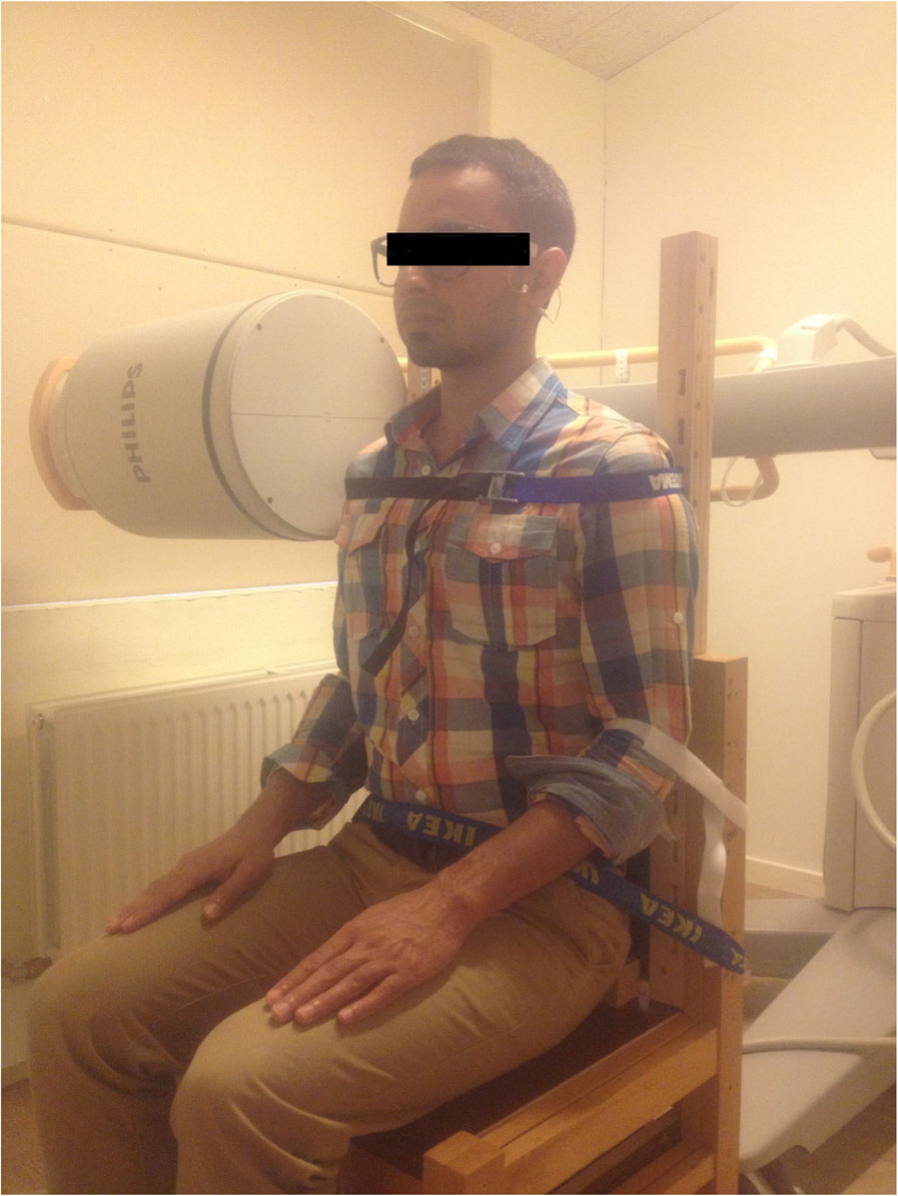
shows the experimental chair with the subject in the sitting position. Straps around shoulders, elbows and waists were used to restrict movements below the cervical spine

Static baseline fluoroscopy images from two repetitive flexion and two repetitive extension motions were extracted from a larger study of cervical dynamic motion. For data acquisition subjects were seated in a chair with hips, knees and ankles at 90°. Shoulders, elbows and waists were fixed by straps (Fig. 1).

Subjects were instructed to sit in their normal upright head and neck position and return to this initial upright head and neck position as precisely as possible after cervical motion and stay in that position for approximately 4 s until beginning of the second repeated motion. The return motion to the upright position was not recorded in order to reduce radiation exposure (Table 1). Timing and execution of experimental tasks were practiced several times before recording, subjects were instructed to move their head and neck through their entire range of flexion or extension and return to the initial upright position. To reduce out of plane motion subjects were provided a line in the sagittal plane on the wall, ceiling and floor. Subjects were instructed visually to follow this line from a mark at their eye height, and return to this mark.

**Table 1:** Experimental procedures

	Flexion 1	Return of flexion 1	Flexion 2	Return of flexion 2	Setup adjustment	Extension 1	Return of extension 1	Extension 2	Return of extension 2
Time interval	16	4	16	4	280	16	4	16	4
Time line	16	20	36	40	320	336	340	356	360
Fluoroscopy video	X		X			X		X	
Reposition Data	0–2		0–2			0–2		0–2	
‘Flexion’	**→**		**←**						
‘Extension’						**→**		**←**	
‘Setup adaptation’			**→**			**←**			
‘Complete Session’	**→**							**←**	

Experiment stages illustrated by rows 1) time interval of the stage, 2) experiment timeline, 3) recording of video (X), 4) the time interval of the stage from where the experiment data was extracted and 5–8) illustration of time periods and experiments stages (→ ←) of the four reposition errors (‘Flexion’, ‘Extension’, ‘Setup adaptation’, ‘Complete Sessions’). ‘Flexion’ and ‘extension’ includes cervical flexion and extension motions with 20 s intervals. ‘Setup adaptation’ includes experimental set up change of 300 s. ‘Complete sessions’ reposition errors between flexion and extension was timed to 340 s. The experimental setup change between flexion and extension motions was timed to approximately 280 s

Two flexion movements followed by two extension motions were recorded from upright to end range. The static position at the beginning of each motion was recorded for 2 s, and the baseline image was extracted from these 2 s. The outcome image was recorded with 4 s delay at the beginning of the second repeated motion (Table 1). Thus the recordings included the baseline positions from consecutive motions. The static images were screened for motion wobble, and the extracted video images represent the static positions found in accordance with the experimental procedures.

The diameter of the fluoroscopic screen was too small to accommodate acquisition of both flexion and extension motions without a 300-s change in the experimental setup. The experimental chair was moved with the subject fixed and without active participation from the subject. Subjects were at the end of change in set up reminded to return to the previously memorized baseline position before data acquisition (Table 1). Subjects were instructed to return to and remain in the memorized upright baseline position through the experimental session. Flexion and extension movements were free and unrestricted. The flexion and extension time was approximately 16 s including 2 s of static imaging at upright and end-range, and after data acquisition the subjects returned to upright at their own pace.

### Fluoroscopic recordings

Fluoroscopy videos (see Additional file 1) were recorded from the upright position to the end range of either the flexion or extension movement at 25 frames per second with an average of 45 KV, 208 mA, 6.0 ms X-ray pulses and average source-to-subject (C7) distance of 92.4 cm. The video sequences were digitalized and stored on a computer. The average radiation dose for the variability study of cervical motion patterns was calculated to be 0.48 mSv by PCXMC, the radiation dose for the static images included in this study is calculated to 0.06 mSv [29].



**Additional file 1:** Fluoroscopic video of cervical motion. A flexion video of cervical flexion movement recorded by fluoroscopic video technology. Find the file at http: (MP4 4148 kb)


### Image analysis

**Fig. 2:**
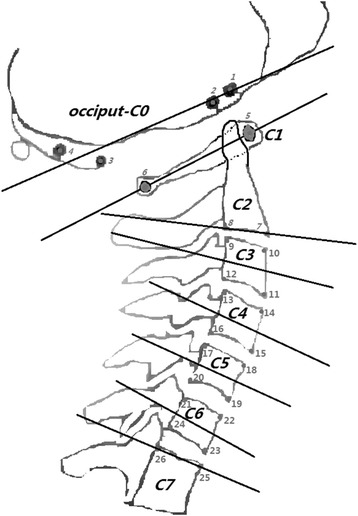
shows the analysis marking points. Four metal balls on pliable wires attached to a pair of glasses served as external markers for occiput (C0), the central areas of the medullary cavities of the anterior and posterior arch were marked on atlas (C1). Two points in proximity to the inferior vertebral plate were marked on axis (C2). The third to the sixth cervical vertebrae (C3-C6) were marked with 4 points in proximity to the vertebral plates. The seventh cervical vertebra (C7) was marked with two points in proximity to the superior vertebral plate. The mid-planes were calculated from the marking points, and joint angles were further calculated between mid-planes. The author XW created the skeletal illustrations

Each image was manually marked and analyzed in a custom-designed Matlab-based program. Calculations of joint rotation were according to a previous validated method [7, 9, 30, 31]. The cervical joint motion was determined from the vertebral midplanes [3, 7]. Change in cervical joint motions were recorded as reposition errors in degrees. The analysis returned individual joint angles in degrees. The midplanes were resolved with respect to the horizontal plane, and change in the midplanes of C0 and C7 demonstrated *a* change in head position and the thoracic spine below C7, respectively. Anatomical structures were marked on all vertebrae except for C0, which were marked with 4 external markers (Fig. 2).

### Reposition error

Data collection yielded four upright baseline positions (flexion1, flexion2, extension1, extension2) for C0/C1, C1/C2, C2/C3, C3/C4, C4/C5, C5/C6, and C6/C7 (Table 1). The upright positions were used to calculate four reposition errors (‘flexion’, ‘extension’, ‘setup adaptation’ and ‘complete session’) as illustrated in Table 1. Reposition errors were analyzed as CEs and AEs. The CEs were the change in joint disc midplanes, and the AEs were the absolute values of the CEs. Previously, constant errors (CEs) and absolute errors (AEs) have been applied in studies of reposition errors [32–35]. CE represents the average magnitude of reposition errors demonstrating multidirectional under and over estimations of target position [33]. The AE represents the average AE and is calculated as the absolute value of CE [33]. The joints moved in extension with positive change and in flexion with a negative change, thus the sign indicates the direction of the errors.

### Statistical analysis

Reposition errors are presented as mean and standard error of measurement (SEM), and measurement errors are presented with SEM. Reposition errors and measurement errors were tested for normality with the Shapiro-Wilk test demonstrating skewness of reposition errors but normal distribution of measurement errors.

For each joint CEs or AEs in the four comparisons (‘flexion’, ‘extension’, ‘setup adaptation’ and ‘complete session’) were compared using the Friedman test and if significant followed by post-hoc assessments by the Wilcoxon test with Bonferroni corrections. Kruskal-Wallis test was performed for difference of CEs or AEs between joints in each task (‘flexion’, ‘extension’, ‘setup adaptation’ and ‘complete session’) and post-hoc Mann Whitney *U*-test with Bonferroni corrections were applied to assess specific differences. The significance level was set at *P* < 0.05.

One investigator (XW) analyzed an upright image three times for intra-rater reliability. Intra-class correlations coefficient (ICC 3,1) assessed reliability of image marking. The minimal detectable change (MDC) for the measurement error was further calculated by 1.96 × √2 × SEM [36]. Statistical analysis was performed in SPSS (IBM Statistics 22).

## Results

In total, 140 cervical spine joints were analyzed among the 20 participants. However, the shoulder shadow in two subjects obscured C5/C6 and C6/C7 yielding 136 joints for analysis. The intra-rater analysis results across joints are presented in Table 2. The intra-rater reliability test demonstrated the average SEM marking error across the three images to be between 0.13° and 0.42°. The ICC of the intra-rater image marking for the three images was 0.998. The reported reposition errors occurred mainly within the C0 to C7 range, as the average changes of C0 and C7 with respect to the horizontal plane across the four upright positions were mean (SEM) 0.53° (1.64°) and 1.04° (1.07°), respectively.

**Table 2:** Minimal detectable change

Joints	C0/C1	C1/C2	C2/C3	C3/C4	C4/C5	C5/C6	C6/C7	Overall
MDC	0.35°	0.70°	0.70°	0.73°	1.17°	1.17°	0.58°	0.73°
SEM	0.13°	0.25°	0.25°	0.26°	0.42°	0.42°	0.21°	0.26°

Minimal detectable change across joints calculated from the standard error of measurement error by 1.96 × √2 × SEM. MDC indicates minimal detectable change. SEM indicates standard error of measurement error

### Constant errors

Table 3 presented CEs from the four tasks. Twelve of 28 joints in Table 3 exceeded the joint MDC in Table 2.

**Table 3:** Constant reposition errors of cervical joint

Joints	Flexion	Extension	Setup adaptation	Complete Session
Average	0.21 ± 0.28	0.01 ± 0.30	0.12 ± 0.45	0.34 ± 0.44
C0/C1	1.74 ± 0.88	1.74 ± 0.68	−4.82 ± 1.98*	−1.35 ± 2.01
C1/C2	0.05 ± 0.79	−0.66 ± 1.20	1.66 ± 1.09	1.06 ± 1.33
C2/C3	−0.70 ± 0.71	−0.52 ± 0.60	1.96 ± 0.67	0.74 ± 0.71
C3/C4	−0.63 ± 0.58	−0.65 ± 0.69	0.25 ± 0.81	0.27 ± 0.54
C4/C5	1.49 ± 0.61	−0.37 ± 0.66	−0.32 ± 0.69	0.80 ± 0.80
C5/C6	−0.31 ± 0.67	0.27 ± 0.79	0.42 ± 0.76	0.38 ± 0.85
C6/C7	−0.29 ± 0.78	−1.14 ± 0.81	1.89 ± 0.98	0.46 ± 1.37

Mean (± SEM) of reposition errors in degrees from tasks defined in Table 1 (‘flexion’, ‘extension’, ‘setup adaptation’ and ‘complete session’). Significantly different from C2/C3 (*, *P* < 0.05)

The average CEs across all joints were small for all tasks (Table 3). Head and neck showed small average CEs the Mean (SEM) of ‘flexion’ and ‘extension’ were 0.21° (0.28°) and 0.01° (0.30°), respectively. The range of all the four tasks *was −*21.1° to 14.3°. The range showed that cervical joints displayed a large variation of repositioning errors at a single joint.

Analysis of differences in CEs within joints was significant for the ‘setup adaptation’ task (Kruskal Wallis, *P* < 0.05), and post-hoc test showed that the C0/C1 reposition error was larger than C2/C3 (*P* < 0.05) (Table 3). Friedman test showed no significant effect of time delay on constant reposition errors.

### Absolute errors

All AEs in Table 4 exceeded the MDC. Average AEs were larger compared to CEs. Absolute ‘flexion’ and ‘extension’ were 2.36° (0.19°) and 2.50° (0.22°), respectively (Table 4).

**Table 4:** Absolute reposition errors of cervical joint

Joints	Flexion	Extension	Setup adaptation	Complete Session
Average	2.36 ± 0.19*	2.50 ± 0.22*	3.31 ± 0.35	3.45 ± 0.33
C0/C1	2.36 ± 0.67	2.34 ± 0.59	5.98 ± 1.80	6.13 ± 1.47
C1/C2	2.50 ± 0.54	2.92 ± 1.00	3.67 ± 0.80	4.06 ± 0.98
C2/C3	2.14 ± 0.54	2.13 ± 0.37	2.60 ± 0.54	2.21 ± 0.52
C3/C4	1.98 ± 0.38	2.49 ± 0.42	2.56 ± 0.57	1.75 ± 0.37
C4/C5	2.19 ± 0.48	2.28 ± 0.41	2.57 ± 0.37	2.94 ± 0.47
C5/C6	2.32 ± 0.37	2.45 ± 0.53	2.37 ± 0.51	2.75 ± 0.53
C6/C7	2.32 ± 0.55	3.01 ± 0.45*	3.29 ± 0.73	4.35 ± 0.89

Mean (± SEM) of reposition errors in degrees across ‘flexion’, ‘extension’, ‘setup adaptation’ and ‘complete session’. ‘Extension’ was different compared with ‘complete session’ (*, *P* < 0.05)

The average AEs found for ‘flexion’, ‘extension’, ‘setup adaptation’ and ‘complete session’ were different from each other (Table 4; Friedman, *P* < 0.05). Post-hoc test showed that the average AEs were smaller for ‘flexion’ (*P* < 0.05) and ‘extension’ (*P* < 0.05) compared to ‘complete session’. The result showed the increased AEs for 340 s time delay (‘complete session); however, a similar increase was not found for 300 s time delay (‘setup adaptation’).

The CEs and AEs of the upper cervical region (C0–C2) showed larger reposition errors compared to the middle and lower cervical regions (Table 3 and Table 4).

## Discussion

The study demonstrates that the position of head and cervical joints varies when repositioned in the memorized upright postures. Twelve out of twenty-eight CEs exceeded the MDC, and all AEs exceeded the MDC. The first study hypothesis was confirmed with respect to AEs, as all single cervical joints demonstrated reposition errors which exceeded the MDC. In contrast the MDC was only exceeded by 12 out of 28 CEs.

The study gave conflicting evidence on the second hypothesis, which tested if reposition errors are increased with longer time delays, as one (340 s AE) of the two long time delays showed significantly different reposition errors, while the other (300 s AE) showed no difference. Likewise, no increased reposition errors were demonstrated with time for CEs.

### Constant errors & absolute errors

The average AE of the flexion movements in this study was 2.36°, similar results were documented by Artz et al. after flexion movement with reposition errors from 1.61° to 2.25° [8]. The CE was also calculated as the average CE across all cervical joints and in ‘flexion’ it was 0.21° ± 0.28° which is in contrast to the larger average AE for all joints (2.36° ± 0.19°) in ‘flexion’.

This study showed large reposition errors for individual joints; however, these large reposition errors were frequently counterbalanced by large reposition errors in other cervical joints in order to acquire a suitable head position. The average CEs were close to zero, this is in line with a previous study of head and neck repositioning, this study found the reposition error suitable for group comparisons within or between patients [4].

Proprioception from muscle spindles is a factor in motor control [37–39] and muscle spindles are more frequent in the upper cervical region compared to the lower cervical region [40]. However, the larger numbers of muscle spindles in the upper cervical region are not reflected in the reposition errors found in the upper cervical region. Larger reposition errors were demonstrated in the upper cervical region compared to the lower cervical region. The results are in agreement with the results found by Treleaven et al. in neck pain disorders [21]. Thus, larger upper cervical region reposition errors have been demonstrated both in healthy subjects and patients with neck pain. Treleaven et al. suggested that upper and lower regions should not be grouped in reposition error studies of whiplash as the grouping may decrease homogeneity. This study supports this suggestion for healthy subjects. This study also suggests that the cervical spine should not be regarded as a single unit of motion, the cervical spine should be regarded as a complex structure with multiple units of motion.

The reposition errors documented in this study appear to reflect the normal variance of cervical motions, and the reposition errors may furthermore be reflected in repeated dynamic neck movements to and from the upright position. The average absolute upright head and neck reposition error (2.36°) may profoundly influence the cervical joint motion pattern, as 2.36° may be a large proportion of the total joint motion.

The results show a variance in reposition of head and neck, the variance may be attributed to normal variance of motor control; however, the variance may also be influenced by other factors such as posture. Forward head posture was associated with larger reposition errors after flexion and extension movements, when compared to a control group [12]. However, the sitting posture did not vary significantly for postural repositioning errors of cervico-thoracic angle compared to control groups [13]. The joint reposition errors demonstrated in this study may improve the understanding of cervical joint position sense. The study suggests the need to control for normal variance of sensor positions in studies of cervical reposition errors, and the study further suggests that results across multiple joints may have varying contributions from those joints.

### Time delay and reposition error

No significant difference of the head and neck was found for CEs of 20 s, 300 s or 340 s time delays. For AEs averaged across joints, twenty seconds delay gave smaller errors than 340 s delay although no difference was found between 20 s delay and 300 s delay.

### Study limitations

The largest confounder was the measurement error and this was reflected in the MDC. The orientation of C0 and C7 with respect to horizontal changed; however, the average change was small. Other confounders, such as image distortion, out of plane motions and natural variance were not reflected in the MDC; however, only upright images were used, so errors due to image distortion and out of plane motion were minimal, and the objective of the study was to assess natural variations. The inter-rater examiner was not blinded, as the marking procedure required the shape of the initial marking of a specific vertebra to be reference for subsequent markings of that vertebra in a marking session. The investigator was not blinded to his own ratings, as the marking procedures required the shape of the initial marking of a vertebra as reference for subsequent markings of that vertebra in a marking session. The study investigated repositioning of single cervical joints after free and unrestricted motion with visual tracking of a line on the floor wall and ceiling. Most reposition studies of the head and neck are conducted blindfolded. The nature of neck pain is recurrent. Thus, no neck pain within the last 3 months may not ensure that all included necks were healthy.

## Conclusion

This is the first study to investigate reposition errors of single cervical joints after flexion and extension movements. The average CEs after flexion and extension movements were 0.21° and 0.01°, respectively. The average AEs after flexion and extension movements were 2.36° and 2.50°, respectively. This results indicated that in healthy subjects the cervical spine returns to the neutral upright position with an error of approximately 2.5° after flexion and extension movements.

## Abbreviations

BMI: Body mass index
SEM: Standard error of measurement
ICC: Intra-class coefficient correlation
MDC: Minimal detectable change
CE: Constant error
AE: Absolute error
